# Evidence of a genomic basis for growth rate variation in a natural kelp population

**DOI:** 10.1038/s41598-026-36286-8

**Published:** 2026-01-29

**Authors:** Samuel Starko, Celina Burkholz, Jane M. Edgeloe, David Wheeler, Karen Filbee-Dexter, Jacqueline Batley, Antoine J. P. Minne, Melinda A. Coleman, Thomas Wernberg

**Affiliations:** 1 School of Biological Sciences, UWA Oceans Institute, University of Western Australia, Crawley, WA Australia; 2 School of Molecular and Life Sciences, Curtin University, Bentley, WA Australia; 3 Department of Primary Industries and Regional Development New South Wales, Coffs Harbour, NSW Australia; 4 Institute of Marine Research, His, Norway; 5 School of Biological Sciences, University of Western Australia, Crawley, WA Australia

**Keywords:** ddRAD, Genomic prediction, GWAS, Heritability, LFMM, Population genomics, Polygenic traits, Reduced representation sequencing, Ecological genetics, Ecophysiology

## Abstract

Understanding the genetic architecture of functional traits can provide key insights into the ecological dynamics and adaptive potential of species. We investigated whether genetic data can predict growth rate variation in a natural population of the widespread kelp, *Ecklonia radiata*. We tagged kelps and tracked their growth in situ over spring when growth is maximal. Individual kelps were then genotyped using reduced representation sequencing (ddRAD) and we employed multiple approaches to assess whether genetic variation corresponded with growth rate variation. Despite a limited sample size, we found evidence that growth rate can be strongly predicted from genetic variation, with approximately half of the variation in growth rate predicted by only 18 loci (R^2^ = 0.499). Leveraging published transcriptomic data, we confirm that most of these loci are expressed or are linked to expressed putative genes. However, many of these genes are of unknown function and do not match well-known gene families. These findings have important implications for understanding natural kelp forest dynamics and for applied approaches such as selective breeding and aquaculture. While our study offers an important first assessment of the possible genomic architecture underlying growth rate in *E. radiata*, future work is needed to confirm this apparent link between genetic and functional variation.

**Supplementary Information:**

The online version contains supplementary material available at 10.1038/s41598-026-36286-8.

## Introduction

Understanding the genetic architecture underlying complex traits in marine organisms is fundamental to both understanding their basic biology and to predicting their adaptive potential in the face of global climate change^1–5^. Growth rate is often directly linked to fitness^6–8^ and it is a key trait that plays a crucial role in shaping population dynamics and ecosystem functioning, particularly in foundation species such as kelps^9,10^. These large brown macroalgae (Laminariales, Phaeophyceae) form the basis of important nearshore habitats that support rich biodiversity and provide numerous ecosystem services to humans^11,12^. Moreover, they form highly productive habitats^13^ and their growth fuels secondary production of higher trophic levels^14,15^. Environmental factors such as temperature, light availability, and nutrient concentrations are known to significantly influence kelp growth (e.g.,^16–18^). However, the extent to which genetic variation contributes to growth rate variability remains a largely open question (but see studies on early life stages^19,20^).

The genetic basis of complex traits often involves several loci of small to moderate effect, a phenomenon known as polygenic inheritance^21–23^. Quantitative genetics approaches which utilize genome-wide markers, have identified the genetic basis for complex traits in various organisms, such as embryophytes and metazoans^23–27^. These methods have been successfully applied in agricultural and forestry contexts^25^, but remain relatively unexplored in marine ecology where they could help deepen our understanding of intraspecific variation in performance and resilience. In the context of kelps, genomic-level data from single nucleotide polymorphisms (SNPs) have begun to elucidate patterns of population structure, phylogeography, and signatures of local adaptation (e.g.,^28–31^), but their application to understand the genetic basis of functional traits remains limited, albeit with great potential.

Applying quantitative genomics approaches to kelps could provide valuable insights into the adaptive potential of these foundation species^32,33^ and the extent to which key traits such as growth rate have a heritable genetic basis. Given the expanding efforts to grow kelp in aquaculture^34^ and for active restoration practices^35^, heritability in growth rate variation would suggest that selective breeding might be an effective means of boosting growth rate and ultimately yield^36^. Yet, while a small number of studies have applied quantitative genomics approaches to test for a heritable basis underlying static traits (e.g., stipe length^37^), the extent to which dynamic traits such as growth rate are genetically determined remains poorly understood. Moreover, while the genes responsible for growth rate variation in other multicellular eukaryotes (e.g., vertebrates, land plants) are increasingly well understood^38–40^, we lack any understanding of what genes may be responsible for controlling growth rate variation in natural populations of brown macroalgae.

One approach that has gained significant momentum over the past decade is utilizing natural populations to screen for genes that control phenotypic variation, an approach sometimes called “wild GWAS”^41^. This approach leverages natural genetic variation to identify potentially adaptive genetic variants without relying on highly controlled experiments and may be particularly valuable in the context of non-model organisms^41^. In the context of kelp, this approach offers potentially promising avenues to identify genetic markers associated with growth rate and other dynamic traits, providing a starting place from which further replication studies and breeding experiments can be designed and conducted.

In this study, we investigated whether genetic variation correlated with growth rate variation in a natural population of the dominant golden kelp, *Ecklonia radiata* (C.Agardh) J.Agardh, in Western Australia. We employed a reduced representation sequencing approach (ddRAD) to generate genome-wide SNP data for tagged kelps (*n* = 52) whose growth rates (measured as lamina elongation rate) were tracked in situ through the austral spring, when growth rates are maximal in this population^42–44^. By combining genome-scale data with field-measured growth rates, we aimed to both provide a first assessment of the potential overall contribution of genetic factors to growth rate variability and identify specific SNPs that may be associated with growth rate variation, potentially revealing key genomic regions involved in growth regulation. Given the logistical challenges of large-scale field phenotyping in subtidal kelp forests, our aim was not to resolve the complete genetic architecture of growth rate or to identify definitive causal loci. Rather, we conducted an exploratory, hypothesis-generating analysis to test whether genomic variation can meaningfully predict field-measured growth variation in a natural population. While we acknowledge that our sample size is limited, this species exhibits substantial inter-individual variation in growth rate^42^, providing an opportunity to evaluate whether variation in particular regions of the genome is strongly associated with this dynamic field-measured trait.

## Results

**Fig. 1:**
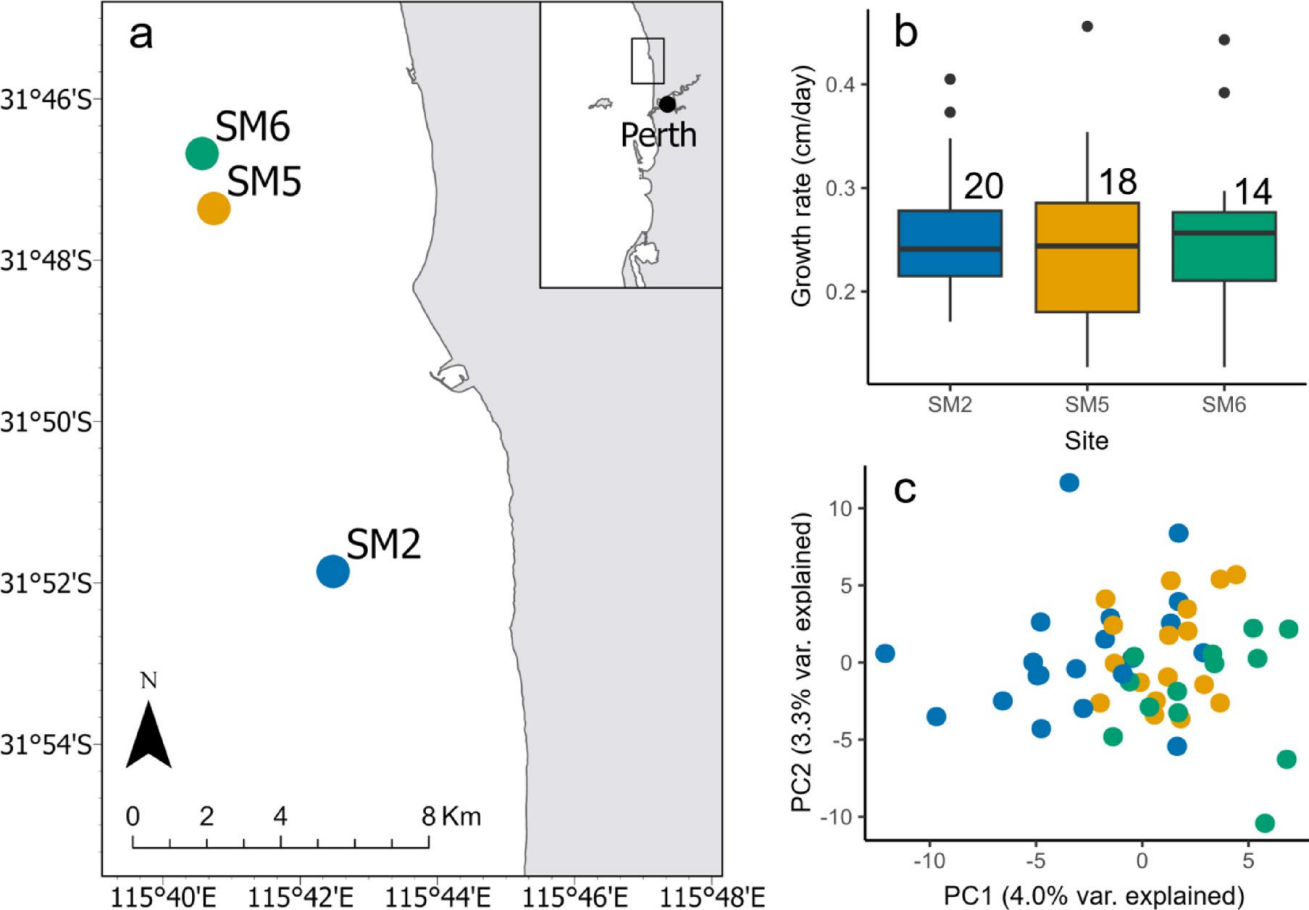
Growth rate and genetic variation of ***Ecklonia radiata*** on Marmion reef. (**a**) Map indicating the location of three sampled sites offshore of Perth, Western Australia. (**b**) Growth rate variation across the three sites showing no significant differences in mean growth rate (ANOVA: *P* > 0.05). Numbers indicate sample sizes for each site. (**c**) PCA displaying genetic similarities inferred from the SNP data. Note overlap in genetic relatedness of individuals across sites.

**Fig. 2:**
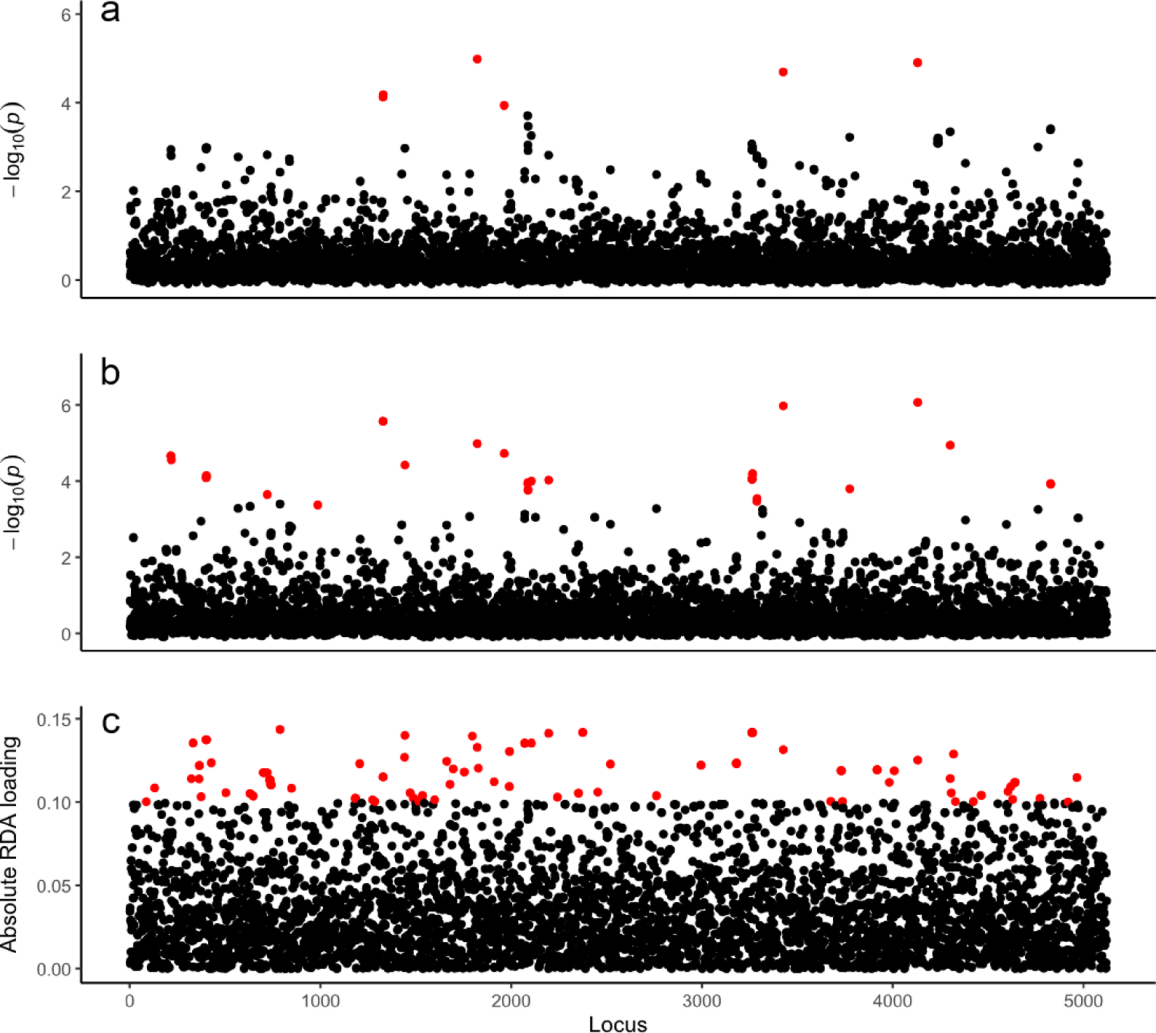
Manhattan plots showing p-values associated with each SNP from (**a**) genome wide association (GWAS), and (**b**) latent-factor mixed effect modelling (LFMM), as well as (**c**) SNP loadings from redundancy analysis (RDA). P-values that are considered significant based on false detection rate thresholds (*P* < 0.05 for LFMM; *P* < 0.10 for GWAS) are shown in red while non-significant p-values are shown in black.

Growth rate varied nearly four-fold across individuals, ranging from 0.12 to 0.46 cm/day. We found no differences in growth rate across sites (ANOVA: F = 0.1707; *p* = 0.8435; df = 51; Fig. 1) or among plots within sites (ANOVA: F = 0.2936; *p* = 0.9643; df = 51) and no relationship between variation in growth rate and overall genetic relatedness of individuals (Mantel test: *p* = 0.866, *r* = −0.0267). However, we detected strong correlations between growth rate and SNP genotype at a relatively small proportion of genetic loci. Using multiple approaches, we identified several individual loci (73 total; ~1.5% of the overall filtered SNP dataset) that had significant associations with growth rate with at least one detection method. Using a genome-wide association (GWAS) as one component of a broader multi-method association analysis, we identified four loci that were significantly associated with growth rate despite the stringent whole-genome false-detection rate threshold of FDR < 0.05. Two additional loci were significant in the GWAS analysis when relaxing the significance threshold to FDR < 0.1 (Fig. 2). LFMM also identified several loci (*n* = 31) that were significantly associated with growth rate (Fig. 2) including the same loci detected using GWAS (*n* = 6; FDR < 0.1) along with an additional set (*n* = 25). Using RDA, we then identified 64 loci that were significantly associated with growth rate, 5 of which overlapped with the significant GWAS loci and 12 overlapped with the significant LFMM loci (Fig S1). To focus on loci for which we were the most confident, we concentrated our analyses on loci that were significant with more than one method (*n* = 18 loci total; ~0.3% of overall SNP dataset).

Given the limited sample size of our study, we conducted a randomization test to determine the probability of detecting spurious loci (i.e., false positives) with GWAS (Fig S2). We found that 92% of the randomizations had only one SNP or less that passed the threshold of FDR < 0.05 (83% had zero). Moreover, less than 2% of the randomizations had 4 or more SNPs that passed this threshold, the number we see in our results. Thus, while limited spurious associations may be possible with our limited sample size, the number of associations that we document here exceeds that which would be expected through random variation.

**Fig. 3:**
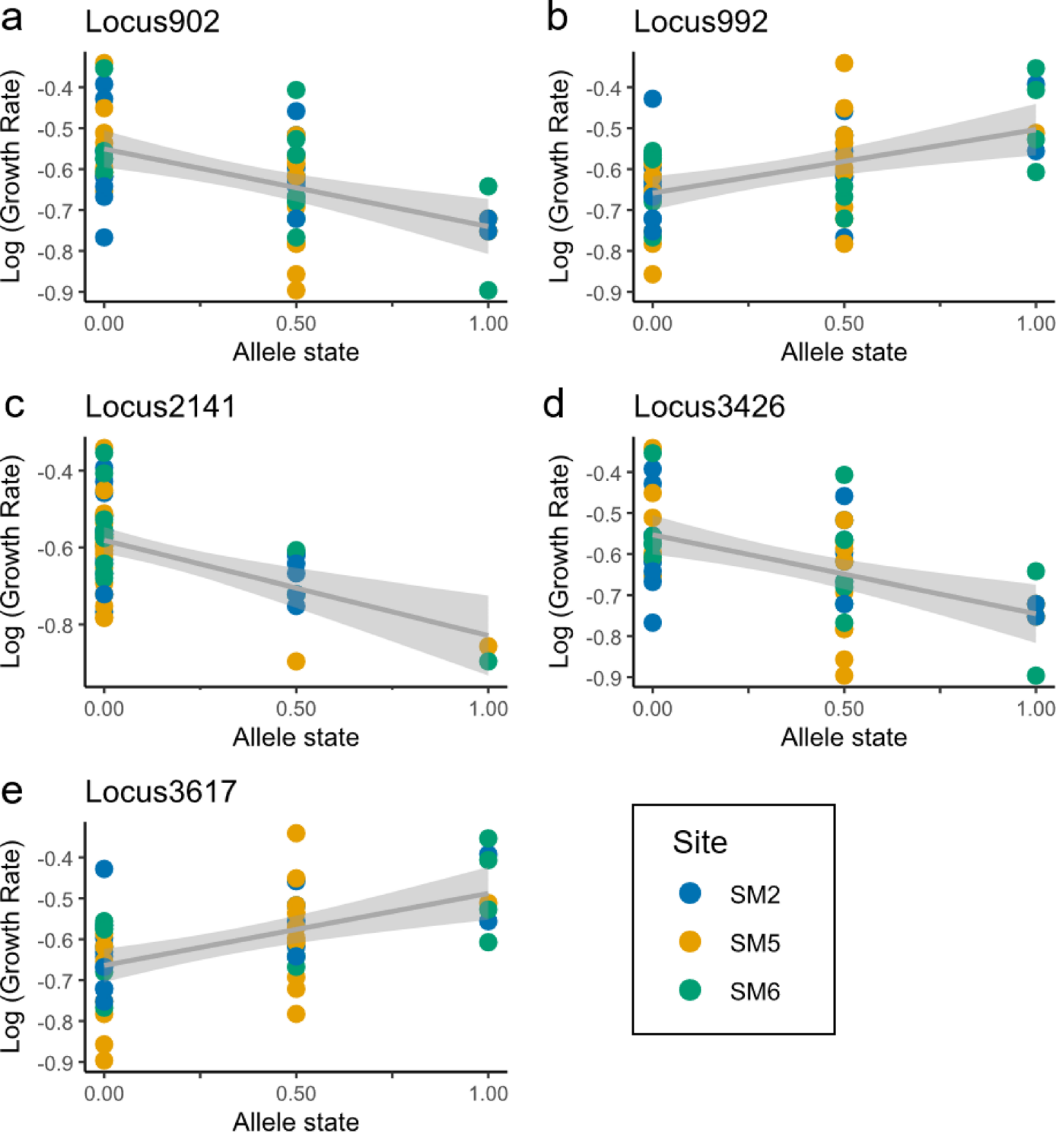
Five loci found to be significant predictors of growth rate based with all three methods used to test for associations, including genome-wide association (GWAS), latent factor mixed effects modelling (LFMM) and redundancy analysis (RDA). (a-e) Each panel shows a particular locus with colour indicating site. A value of zero indicates the individual is homozygous for the major allele, a value of 1 indicates the individual is homozygous for the minor allele and a value of 0.5 indicates a heterozygote. Regression line and confidence intervals are best-fit linear relationships.

**Fig. 4:**
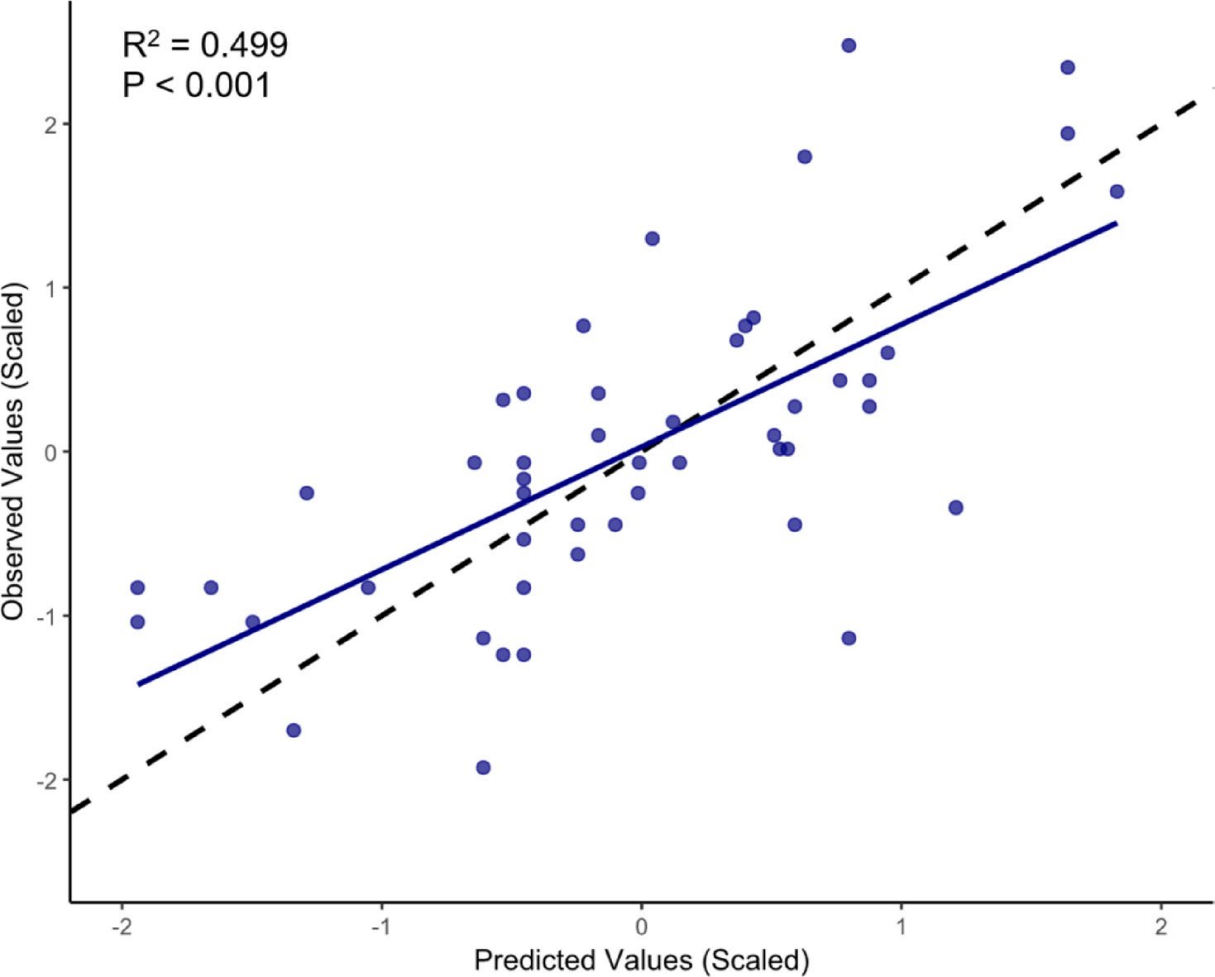
Relationship between predicted and observed scaled growth rate based on LFMM modelling of the 18 loci identified using at least two methods. Dotted line indicates a 1:1 line while the blue line is the best-fit linear regression. Coefficient of determination (R^2^) is also shown.

We identified five SNPs that were significantly associated with growth rate across all three detection methods (GWAS, LFMM and RDA, hereafter “triple-concordant”). Two of these SNPs (55183:7, 55183:146) occurred on the same ddRADtag, indicating that these five SNPs represent four independent genomic regions with strong cross-method support (Table 1). Each of these SNPs explained 23–30% of growth variation individually. Both major and minor alleles were found at each field site for all five of these loci and there was no significant site-by-growth rate interaction, indicating consistency across sites (Fig. 3; ANCOVA results in Table S1). Thirteen additional SNPs were found to be double-concordant (i.e., consistently growth-associated across at least two methods), resulting in 18 SNPs that were significant with at least two detection methods. Each of these 18 SNPs explained ~ 15–30% of growth rate variation individually (Table S2, S3), while the broader set of 18 SNPs explained ~ 50% of variation when combined into a polygenic model (LFMM ridge: R^2^ = 0.4993; *P* < 0.001; Fig. 4).

**Table 1:** Description of the five loci associated with growth according to all three methods (GWAS, LFMM, RDA), indicating the presence of linked gene models and whether the sequence or linked gene is found in the *E. radiata* transcriptome. Coefficient of determination is derived from a linear model fit between allele state and growth rate. Transcriptomic support columns indicate whether there is a match between the ddRADtag sequence on which the SNP is found or the gene model(s) linked to this the ddRADtag locus and the *E. radiata* transcriptome. The closest BLASTx hit for each linked gene model or ddRADtag sequence is also given where a match was found. *Locus 3426 was not found in the transcriptome but has overlap with locus 102,109 which did match the transcriptomes.

Locus ID	Coefficient of determination (*R*^2^)	Transcriptomic support (ddRADtag)	Linked gene model	Transcriptomic support (linked gene model)	Top blastX match
4131 (157782:213)	0.278	No	scf7180001285411.1	Yes	None
			scf7180001285411.2	Yes	LRR-GTPase of the ROCO family [*Ectocarpus siliculosus*]
			scf7180001285411.3	Yes	LRR-GTPase of the ROCO family [*Ectocarpus siliculosus*]
			scf7180001285411.4	Yes	None
1822 (86819:217)	0.275	No	scf7180001330130.1	Yes	Caffeoyl-CoA O-Methyltransferase [*Ectocarpus siliculosus*]
1328 (55183:7)	0.236	Yes	None	-	-
1329 (55183:146)	0.236	Yes	None	-	-
3426 (189431:210)	0.229	No*	None	-	-

Across the 18 multi-method SNPs, 12 distinct ddRADtags (contig sequences generated from the ddRAD data) carried significant variants, including five SNPs that were clustered on a single ddRADtag, indicating localized linkage among some markers. A majority of these ddRADtags (7 of 12; ~58%) either mapped directly to the *E. radiata* transcriptome (Sequence Read Archive: SRR3709347) or were linked to nearby gene models in the draft *E. radiata* genome (see Methods) that were themselves expressed in the transcriptome. Five ddRADtags (~ 45%) were associated with 12 annotated gene models when comparing against the *E. radiata* draft genome. However, only two gene families with recognised biological functions were represented among these: LRR-GTPase genes from the ROCO family (which were linked to two growth-associated ddRADtags), and Caffeoyl-CoA O-Methyltransferases which were linked to the two most strongly predictive SNPs (Table 1, S2, S3). The remaining six gene models linked to double- or triple-concordant SNPs were annotated as proteins of unknown function. Of the ddRADtags that lacked links to gene models (*n* = 7), five had transcriptomic matches but no identifiable BLASTx hits in the NR protein database, suggesting these loci represent expressed but currently uncharacterized brown-algal genomic regions that may play biologically meaningful roles.

## Discussion

Understanding the genetic basis and heritability of traits, such as growth rate, in kelp is critical for aquaculture, restoration and assisted adaptation approaches^33^, but such knowledge lags behind other taxa^32^. Our study suggests that growth rate variation in *Ecklonia radiata*, one of the most widespread species of kelp globally^45^, is influenced in part by genetic variation. This finding represents the first evidence of a genetic component (R^2^ = 0.499 for 18 double- or triple-concordant loci combined) underlying a key physiological trait in a natural kelp population, with potential implications for both evolutionary ecology and applications like aquaculture and assisted adaptation^33,46^. The identification of five growth associated loci that were consistent across all three methods (GWAS, LFMM and RDA) and 13 additional loci that were significant using at least two of these methods, suggests several specific genomic regions that contribute meaningfully to growth rate variation.

This potentially strong genetic component has several important implications. First, it suggests that growth rate in *E. radiata* may be a strongly heritable trait, potentially lending important insights into the factors driving growth variation in natural kelp populations. Second, from an applied perspective, our results have important implications for kelp aquaculture. The strong genotype-phenotype associations we identified suggest that selective breeding programs could effectively enhance growth rates in cultivated kelp. However, given the potential for complex interactions between different genes and their influence on dynamic traits^47,48^, future work is needed to determine the extent to which causal loci have additive or interactive effects on growth rate. Nonetheless, this finding may be important given the growing interest in kelp aquaculture for both food production and other ecosystem services^34,49^. Nonetheless, recognizing heritable variation in traits such as growth could improve donor selection in restoration and assisted adaptation, potentially maximizing both productivity and climate resilience^33,36^.

In general, growth in indeterminant, modular taxa is often considered an emergent trait resulting from several interacting physiological and ecological processes related to resource acquisition and allocation^23,53^. Thus, it is often expected that growth rate variation should be driven by a large number of genomic loci, each with relatively small effect^23,53^. Yet studies on tree growth have generally found that a relatively small number of loci are responsible for growth rate in land plants. For example, a study on *Populus* found that nearly half of the variation in stem growth rate could be explained by just two loci^54^ and a small number of loci appear to drive growth rate variation in *Eucalyptus*^55^ and *Pinus*^56^ with individual loci explaining similar amounts of variation to the most strongly growth-associated markers from our study (e.g., ~ 20%). However, other work has found smaller effects of individual loci (e.g., in *Picea abies*^57^) suggesting that these patterns vary by taxa or by conditions. Our results fall between these extremes, indicating that growth rate under the environmental conditions sampled here may be influenced by a handful of loci of moderate effect plus several of smaller effect.

It is important to note that the limited sample size of our study (*n* = 52) likely influenced our power to detect growth-associated loci. Smaller sample sizes in quantitative genomic analyses (especially GWAS) can increase both false positives and false negatives^58,59^. However, our randomization test showed that detecting four significant loci with GWAS (FDR < 0.05) was more extreme than > 98% of randomizations, suggesting that the most strongly supported associations are unlikely to be spurious. Moreover, the lack of any interactions between site and genotype suggests that there are similar associations between growth and allele state across different sites, a pattern we would only expect when genetic effects are stronger than those of site-level environmental variation.

The presence of both major and minor alleles at all study sites, with no significant effect of site, indicates that this species maintains substantial standing genetic variation for growth rate, at least across the area of reef sampled. It remains unclear why such variation would remain within the population and not be fixed over time if fast growth were adaptive (i.e., led to increased fitness). This could suggest balancing selection^60^, perhaps mediated by spatial or temporal variation in optimal growth rates, or trade-offs with other fitness-related traits, including traits expressed in the microscopic gametophyte stage. One possibility is that the drivers of growth depend on the environment. For example, while we measured growth during optimal conditions (i.e., late winter into spring), Marmion reef experiences temperatures in summer months that exceed thermal optima for *E. radiata*, and these warmer conditions are associated with reduced growth rates overall^16,61^. It is possible that fast growth in the spring is traded off against other traits such as thermal tolerance, which may be important in the summer or during extreme warming events (i.e., marine heatwaves). Future work should test how these genomic predictors of growth rate vary across different environmental conditions (e.g., seasonally or across latitudes) and their potential link with other traits such as thermal tolerance. Indeed, growth rate has been shown to negatively correlate with environmental tolerance in some photosynthetic taxa (e.g^62^.,). Alternatively, given that optimal growth in this population of *E. radiata* co-occurs with the largest storms and swell events^44^, rapid growth to large sizes may be associated with increased risk of dislodgement^63,64^, potentially offsetting any fitness advantage from outgrowing other conspecific individuals. Critical to understanding these potential trade-offs is an understanding of which genes or molecular pathways drive variation in growth rate.

Several growth-associated SNPs were physically linked to genes in the draft genome of *E. radiata* or matched transcribed sequences. Even in cases where the SNP itself was not transcribed, close linkage to expressed genes suggests that these loci could play a role in modifying gene expression (e.g., as regulatory variants^65^) rather than altering protein structure. Alternatively, these loci themselves may be neutral but they could be associated with causal variants through linkage disequilibrium. Interestingly, in most cases these transcribed sequences were either associated with genes of unknown function or lacked an identifiable gene within the transcript, highlighting gaps in our functional understanding of brown macroalgal genomes^66^. Interestingly, the two loci most strongly associated with growth rate were linked to gene models from known protein families with plausible links to growth rate (cell wall metabolism and signalling). The most strongly associated locus was linked to a Caffeoyl-CoA O-Methyltransferase gene, which has documented roles in structural and metabolic processes in land plants including lignin biosynthesis^69^. The potential involvement of this gene in kelp growth is intriguing, suggesting parallels in the molecular mechanisms underlying growth across plant-like lineages. While brown algae are not lignified, polyphenolic components are central components of brown algal cell walls^70,71^ and may depend on these types of Methyltransferase genes. These findings underscore the need for further functional studies to clarify the roles of these candidate genes and associated pathways in regulating growth in *E. radiata*.

The second most strongly associated locus, locus 4183 (ddRADtag: 157782; position: 213), was closely linked to two proteins from unknown families and two ROCO-family LRR GTPases, a gene family that also matched one of the double-concordant loci (see Table S2). ROCO proteins are more abundant in brown algae than in any other eukaryotic group and are believed to play diverse signalling roles^67^. In other eukaryotes, ROCO proteins are implicated in processes ranging from growth regulation in *Arabidopsis* to familial Parkinson’s disease in humans^68^. While it has been hypothesized that ROCO genes may play roles in the innate and adaptive immunity of brown algae, our findings suggest that they can be involved in growth variation, potentially through signalling pathways or regulation by nearby promoters or inhibitors. Given the diversification of the ROCO gene family within brown algae, these genes likely fulfill a range of signalling functions^67^, with growth regulation now being one candidate role.

Although we focused on loci supported by two or more methods, several single-method SNPs also displayed relatively strong correlations with growth rate (~ 15–25% of growth variation explained; Tables S2, S3). Many of these were located on ddRADtags that matched the *E. radiata* transcriptome or were positioned within scaffolds containing expressed gene models. Several additional annotated gene families were represented among these single-concordant loci, including predicted proteins with putative roles in signal transduction and cell-wall metabolism (Table S3). These loci may therefore represent additional components of the growth-regulatory network that fell below our multi-method confidence threshold due to limited statistical power, rather than biological irrelevance. As such, they remain promising targets for functional follow-up once larger datasets or controlled experimental designs become available.

While we identified a strong genetic effect (approximately 50% in growth rate variation) across only a handful of loci, there remains substantial unexplained variation in growth rate in our study. A few factors likely contribute to this unexplained variation. While there were no broad site level differences in growth rate, microhabitats can vary along the reef leading to differing environmental parameters such as irradiance levels and wave exposure, as well as both inter- and intraspecific competition with other macroalgae. Moreover, while we aimed to target kelp from a similar developmental stage (mature adult kelp), there was undoubtedly variation in the ages of different kelps which may influence their growth rates and contribute additional unexplained variation. There also could have been additional genetic effects that we failed to detect. RADSeq methods only capture a small percentage of the overall genome making it possible that we missed genomic regions associated with growth rate variation. Alternatively, growth could be controlled by interactive effects of multiple genes^72^ which are missed by GWAS, LFMM and RDA approaches that only model additive effects.

While our findings indicate that a subset of genomic regions are strongly associated with variation in growth rate under the environmental conditions sampled here, we currently lack direct evidence of the causal mechanisms by which these loci influence growth. The functions of many linked genes remain poorly understood in brown algae, and it is not yet known whether their effects are additive, dominant, or shaped by epistatic interactions among loci. These uncertainties mean that our study cannot yet fully assess whether selective breeding programs would achieve sustained gains across generations. Our results are therefore best viewed as identifying candidate genomic regions that warrant functional validation (e.g., expression analyses, controlled breeding, or gene perturbation approaches) before being implemented in applied breeding or assisted adaptation strategies.

Our findings nonetheless open several promising avenues for future research. First, functional studies of the identified genomic regions could both confirm the role of the growth-associated loci and reveal specific mechanisms controlling growth rate variation. While we identified candidate regions, the exact mechanisms or pathways linking these transcribed regions to growth rate variation remain unclear. Comparing gene expression profiles of fast versus slow-growing genotypes may lend insights into the molecular processes that control growth. Second, investigating these loci across the species’ range could uncover how their allele frequencies vary with environmental conditions, potentially revealing signatures of local adaptation and better understanding how growth rate variation plays into the adaptive landscape of kelp populations. Studying how these growth-associated loci interact with environmental stressors such as temperature could provide crucial insights into the species’ capacity to adapt to climate change. Finally, interfering with the expression of candidate growth variation genes (e.g., using RNAi or CRISPR/Cas9 approaches) would unequivocally determine whether these genes are indeed driving growth variation.

In conclusion, our finding that genetic markers strongly predicted growth rate variation in *E. radiata* provides important insights into how trait variation is determined in a critically important foundation species. These findings contribute to our growing understanding of the genomic basis of adaptation in marine organisms and highlight the value of combining field-based phenotyping with modern genomic approaches in marine ecology. Moreover, our findings have important implications for crop breeding in the rapidly growing kelp aquaculture industry, indicating that selective breeding may be particularly effective for producing kelp crops with desirable dynamic traits. However, future work is needed to better understand the role of individual loci and potential interacting effects before implementing such approaches.

## Methods

### Kelp tagging and monitoring

Individuals of *Ecklonia radiata* were tagged at three subtidal reef sites < 10 km apart within Marmion Marine Park, Western Australia (Fig. 1) as part of a larger effort to tag and track growth rate variation^42^. Within each site, three plots (diameter = 5 m) were established that were 10–30 m apart and adult individual sporophytes were haphazardly tagged and tracked within each plot (minimum of 0.5 m apart). All individuals were growing between 8 and 13 m depth and were sampled via SCUBA diving. Growth rate was measured in the austral spring between August 2021 and November 2021, capturing the season of maximal growth^43,44^ when sea temperature is largely stable (Fig S3). Due to both natural mortality and limitations associated with subtidal work on SCUBA (e.g., visibility, no-decompression times), not all tagged kelps could be relocated. However, several individuals (*n* = 3–10) were successfully monitored within each plot across a 79-day period. All sampling of *Ecklonia radiata* was conducted with appropriate permits from the Department of Primary Industries and Regional Development (DPIRD) as well as the Department of Biodiversity, Conservation and Attractions (DBCA).

Lamina elongation rate was measured using the hole-punch method^44,73^ which takes advantage of the fact that most lamina growth occurs in the first 5 cm^16^. A hole was punched into the lamina at a standardized distance from the meristem (5 cm), and the distance that the hole moved distally (upwards) over time was measured. The elongation rate (cm day^− 1^) was calculated based on the distance of the hole from the meristem (measured distance minus 5 cm) divided by the number of days between measurement points (*n*= 79 days). Although variation in elongation rate may not perfectly reflect variation in productivity from a biomass perspective and may miss changes in thickness or width of individuals, it is a necessary and widely applied (e.g.,^16,74^) approach when measuring growth non-destructively. Moreover, there tend to be strong relationships between length and overall biomass in kelps^75–77^, suggesting that elongation can still offer substantial insight into overall rates of productivity.

The distribution of growth rate data deviated from normality due to a slight right skew (Shapiro-Wilk Test: W = 0.9459; *p* = 0.0181). However, following log_10_-transformation, the data were normally distributed (Shapiro-Wilk Test: W = 0.9838; *p* = 0.6969; Fig S4). Thus, we applied this transformation to the growth measures for all analyses to meet the assumption of normality.

We measured ambient seawater temperature (°C) throughout the monitoring period at each study site. Temperature loggers (HOBO TidbiT v2 UTBI-001 or HOBO MX2203 TidbiT; *n* = 1 per site) were deployed at canopy height and recorded temperature hourly throughout the period of the study.

### DNA extraction, library Preparation and sequencing

Small samples of laterals from tagged kelp were collected in the field and rapidly frozen in liquid nitrogen to preserve DNA integrity. Frozen tissue was then ground with a tissue lyser and total genomic DNA was extracted using the Qiagen DNeasy Plant Mini Kit (Qiagen, Germany) with a modified protocol following Vranken et al.^78^. Incubation time was extended to 48 h and an additional wash of 95% ethanol was conducted prior to elution of DNA from the spin column. We then performed a clean-up on the DNA before library preparation using the Qiagen Powerclean kit (Qiagen, Germany). DNA concentration was quantified with a Qubit fluorometer using the dsDNA Broad Range assay (Invitrogen) and quality was assessed using either a LabChip GX Touch 24 (PerkinElmer) or a standard gel electrophoresis.

In total, 200ng of DNA per sample was used to prepare ddRAD (double digest restriction associated DNA) libraries by the Australian Genome Research Facility (AGRF) following the protocol of Severn-Ellis et al^79^. with some modifications, specifically using restriction enzymes *Pst*I and *Nla*III. Following digestion with restriction enzymes (New England Biolabs), unique barcoded adaptors were ligated to DNA fragments from each sample. Double size selection was performed using SPRI beads (AMPure XP, Beckman Coulter) to retain fragments between 250 and 800 bp. Libraries were amplified using polymerase chain reaction (PCR) and purified using SPRI beads. Sequencing was performed on an Illumina Novaseq X platform (Paired-end 2 × 150 bp), with equimolar concentrations of 48 individuals included across 6 pooled libraries. Each library was composed of up to 48 individuals as part of a collaborative sequencing effort supporting multiple projects.

### SNP obtention and filtering

Paired-end reads were demultiplexed using STACKS (2.64) process_radtags^79^ with quality filtering (-q, -c), barcode rescue (-r), and RADtag checks (--renz_1 pst*I*; -- renz2 N*I*a*III*) performed following protocol described in Vranken et al^78^.. Adapter trimming was performed using TRIMMOMATIC with the parameters ILLUMINACLIP: TruSeq3-PE.fa:2:30:10 before all reads were trimmed to 140 bp with BBMAP (BBMap - Bushnell B. - sourceforge.net/projects/bbmap/T). The final quality of sequenced reads was assessed using FASTQC^80^ and MULTIQC^81^. *De novo* stacks assembly and SNP calling was conducted using the denovo_map pipeline. A minimum distance of three nucleotides was chosen to identify a stack (-m) and a maximum distance of three nucleotides was permitted between stacks in a locus (-M). A total of three mismatches were allowed between orthologous loci of different individuals during catalogue construction (as in^31^). VCFTOOLS version 0.1.16^83^ was used to remove all indels and sites with more than two alleles. Moreover, we set a minimum coverage depth of 5x and a maximum coverage of two-times-mean sequencing depth (mean depth = 27.5). We removed sites with more than 10% missing data and with a minor allele frequency of less than 0.03. All individuals had data for 90% or more of loci thus we did not apply an additional filter to remove individuals based on missing data. We calculated the SNP error rate between technical replicates (i.e., multiple libraries produced and sequenced from the same DNA extract) and found it to be less than 1% in all cases. The resultant dataset included 5,121 SNPs for 52 individuals each of which had associated growth data.

### Statistical analysis

Subsequent analyses were conducted in R 4.3.2 (R Core Team, 2021) using a suite of packages. To manipulate data and convert datasets between different formats, we used the following packages: vcfR^83^, tidyverse^84^, dartR^85^, vegan^86^, snprelate^87^, bigsnpr^88^ and ape^89^. We first tested for normality in the growth rate dataset using a Shapiro-Wilk test (using the Shapiro.test function in base R). We then calculated genetic relatedness of individuals using the A.mat function in rrBLUP^90^ and conducted a Mantel test (using the base package in R) to test for a correlation between genetic relatedness and inter-individual differences in growth rate.

Because sample size can strongly influence power and false discovery rates in association analyses, particularly in non-model organisms, we adopted a deliberately conservative, multi-method framework. Rather than relying on a single GWAS approach, we combined GWAS, LFMM, and redundancy analysis (RDA), and focused interpretation on loci supported by two or more methods. This approach is increasingly recommended for exploratory genotype–phenotype analyses when sample sizes are necessarily limited and serves to reduce the likelihood of spurious associations. First, we performed a genome-wide association (GWAS) analysis correcting p-values for false detection rate (FDR). GWAS involves fitting a series of linear models between growth rate and the allele state of each SNP and this was performed in bigsnpr^88^. P-values were then corrected to account for multiple comparisons using qvalue^91^. Because Principal Component Analysis (PCA) showed very limited structure across the different sites (Fig. 1C) and there was no significant effect of genetic relatedness and differences in growth rate (based on the Mantel test), we did not correct for genetic relatedness in our GWAS analysis. Given the high stringency associated with correcting for so many comparisons, and the likelihood of type II error^92,93^, we evaluated loci at significance values of both FDR < 0.05 and FDR < 0.1. Given the low sample size in our study, we performed randomization tests to determine the probability of a SNP being significantly associated with growth according to GWAS at random (i.e., a spurious association). We performed 1000 iterations where w randomized growth rate and then conducted GWAS, noting the number of significant SNPs, generating a null distribution with which to compare our own data.

Latent factor mixed models (LFMM) were also employed in the R package lfmm^94^ to further identify SNPs associated with growth rate while accounting for genetic relatedness of individuals. Although LFMM accounts for genetic relatedness of individuals, it is generally less conservative than GWAS and is therefore a useful approach when a trait is driven by several loci with small individual effects. Moreover, lfmm is generally considered an informative approach when sample sizes are low^94,95^. We used the “ridge regression” option in LFMM, though preliminary testing with the “lasso” option revealed similar results.

We also used redundancy analysis (RDA) as a third method of identifying potentially growth-associated loci. Past work has demonstrated that RDA tends to perform better than GWAS or LFMM when sample sizes are low (e.g., *n* = 50 or below)^58,95^. We conducted this analysis in vegan^86^ and applied a ± 2.5 SD cut-off on the SNP loadings to identify candidate growth-associated loci^31,96^.

To test the overall contribution of significant SNPs associated with growth rate, we calculated a scaled score of the genomic coefficients (similar to a polygenic risk score) and tested how strongly this predicted the observed growth rates by fitting a linear model. The scaled score was calculated by refitted a lfmm ridge regression using the 18 loci that were identified as growth-associated with at least two detection methods. The predictive accuracy of the model was assessed using the coefficient of determination (R²) from a linear model between observed and predicted scaled growth values.

For SNPs that significantly predicted growth rate with any of the three methods (GWAS, LFMM and RDA), we used the contig sequences output from STACKS (i.e., ddRADtag sequences) to determine whether these loci fell within or nearby putative genic regions. We mapped ddRADtag sequences to a publicly available transcriptome for *Ecklonia radiata*^97^ (Sequence Read Archive: SRR3709347) in Geneious Prime 2022.2.2 ^100^, using default settings for mapping sequences to a reference, in order to determine whether the ddRADtag sequence is completely or partially expressed as RNA, which may be indicative of a functional role of this sequence. Because a chromosome-level reference genome is not yet available for *Ecklonia radiata*, linkage between growth-associated SNPs and nearby genes on the genome was inferred based on physical proximity. ddRADtag sequences containing significant SNPs were mapped to a draft *E. radiata* genome (unpublished; available upon reasonable request; see^31^) using BLASTn, with a top hit scaffold selected based on a minimum nucleotide identity of > = 96% to the stacks sequence^31^. All annotated gene models located on the same scaffold and within 50 kb were considered potentially linked. This scaffold-level approach provides a conservative assessment of linkage and is commonly used in reduced-representation genomic studies of non-model organisms. We provide information on the distance between the annotated gene and each ddRADtag hit in Table S3. The Gene Feature format (GFF) file was used to identify all gene models found on candidate scaffolds and these were annotated based on BLASTx homology (e-value < 1e-5) to genes from *Ectocarpus siliculosus* (Dillwyn) Lyngbye^99^. We also aligned these sequences against the transcriptome (using Geneious Prime) to provide additional support that these genes are expressed and therefore potentially functional.

To determine whether growth rate varied significantly across sites, we performed a one-way ANOVA. We also tested for interactions between allele state at a particular locus and site using an ANCOVA. Both analyses were conducted in base R.

## Supplementary Information

Below is the link to the electronic supplementary material.

Supplementary Material 1

Supplementary Material 2

## Data Availability

Raw sequence reads are available on the NCBI Sequence Read Archive (SRA) under BioProject ID: PRJNA1368432. The processed datasets generated and/or analysed during the current study are available on Figshare, [http://doi.org/10.6084/m9.figshare.30528407](http:/doi.org/10.6084/m9.figshare.30528407).
