# Supplementary material for: Evidence of a genomic basis for growth rate variation in a natural kelp population
Source: Sci Rep. 2026 Jan 29;16:6622. doi: 10.1038/s41598-026-36286-8 (PMC12913910; doi:10.1038/s41598-026-36286-8)
Supplement: Supplementary file 1 — Supplementary Material 1 [file 41598_2026_36286_MOESM1_ESM.docx]

**Supplementary Information**

**Evidence of a genomic basis for growth rate variation in a natural kelp population**

Samuel Starko, Celina Burkholz, Jane M. Edgeloe, David Wheeler, Karen Filbee-Dexter, Jacqueline Batley, Antoine J. P. Minne, Melinda A. Coleman & Thomas Wernberg

**Supplementary Tables**

**Table S1. Results of ANCOVAs testing for interactions between allelic status and site for the five loci found to be significantly associated with growth rate using all three methods (GWAS, LFMM, RDA).**

| **Locus ID** | **Allelic status** | **Site** | **Allele status x Site Interaction** |
| --- | --- | --- | --- |
| 1328 | F = 16.2574  **P = 0.0002*** | F = 0.3918  P = 0.6780 | F = 0.8903  P = 0.4175 |
| 1329 | F = 16.2574  **P = 0.0002*** | F = 0.3918  P = 0.6780 | F = 0.8903  P = 0.4175 |
| 1822 | F = 18.6985  **P < 0.0001*** | F = 1.5416  P = 0.2251 | F = 0.6157  P = 0.5447 |
| 3426 | F = 13.2584  **P = 0.0007*** | F = 0.4099  P = 0.6663 | F = 0.3828  P = 0.6842 |
| 4131 | F = 17.8173  **P = 0.0001*** | F = 1.3891  P = 0.2605 | F = 0.7842  P = 0.4631 |

**Table S2.** Description of the 18 growth-associated loci that were detected with at least two methods, indicating the presence of linked gene models and whether the sequence or linked gene is found in the *E. radiata* transcriptome. Asterisks in first column indicates the loci that were significant with all three approaches (GWAS, LFMM, RDA). Coefficient of determination is derived from a linear model fit between allele state and growth rate. Transcriptomic support columns indicate whether there is a match between the ddRADtag sequence on which the SNP is found or the gene model(s) linked to this the ddRADtag locus and the *E. radiata* transcriptome. The closest blastX hit for each linked gene model or ddRADtag sequence is also given where a match was found. More information on each locus as well as all loci detected using any method is found in Table S3 and S4 which are included as CSVs.

| Locus ID | Coefficient of determination (R^2^) | Transcriptomic support (ddRADtag) | Linked gene model | Transcriptomic support (linked gene model) | Top blastX hit |
| --- | --- | --- | --- | --- | --- |
| 3426 (157782:213) | 0.2376 | No | scf7180001285411.1 | Yes | NA |
| 3426 (157782:213) | 0.2376 | No | scf7180001285411.2 | Yes | LRR-GTPase of the ROCO family [*Ectocarpus siliculosus*] |
| 3426 (157782:213) | 0.2376 | No | scf7180001285411.3 | Yes | LRR-GTPase of the ROCO family [*Ectocarpus siliculosus*] |
| 3426 (157782:213) | 0.2376 | No | scf7180001285411.4 | Yes | NA |
| 4131 (189431:210) | 0.2281 | No | NA | No* | NA |
| 1328 (55183:7) | 0.2389 | Yes | NA | No | NA |
| 1329 (55183:146) | 0.2389 | Yes | NA | No | NA |
| 1822 (86819:217) | 0.304 | Yes | scf7180001330130.1 | Yes | Caffeoyl-CoA O-Methyltransferase [*Ectocarpus siliculosus*] |
| 1963 (93598:120) | 0.2243 | No | scf7180001299918.2 | No | hypothetical protein Esi_0006_0207 [*Ectocarpus siliculosus*] |
| 1963 (93598:120) | 0.2243 | No | scf7180001299918.1 | No | hypothetical protein Esi_0006_0207 [*Ectocarpus siliculosus*] |
| 2196 (102109:46) | 0.1756 | Yes | NA | No | NA |
| 3264 (152316:175) | 0.1878 | No | NA | No | NA |
| 3265 (152316:210) | 0.1878 | No | NA | No | NA |
| 3261 (152316:28) | 0.1878 | No | NA | No | NA |
| 3262 (152316:71) | 0.1878 | No | NA | No | NA |
| 3263 (152316:85) | 0.1878 | No | NA | No | NA |
| 402 (15755:104) | 0.1634 | Yes | NA | No | NA |
| 401 (15755:18) | 0.1634 | Yes | NA | No | NA |
| 4301 (195169:163) | 0.2159 | Yes | NA | No | NA |
| 721 (29164:66) | 0.1514 | No | scf7180001375818.1 | No | NA |
| 1443 (64020:196) | 0.1769 | No | scf7180001285411.1 | No | NA |
| 1443 (64020:196) | 0.1769 | No | scf7180001285411.2 | No | LRR-GTPase of the ROCO family [*Ectocarpus siliculosus*] |
| 1443 (64020:196) | 0.1769 | No | scf7180001285411.3 | No | LRR-GTPase of the ROCO family [*Ectocarpus siliculosus*] |
| 1443 (64020:196) | 0.1769 | No | scf7180001285411.4 | No | NA |
| 2106 (98637:70) | 0.18 | Yes | NA | No | NA |

**Table S3. Genomic, transcriptomic and functional annotation metadata for SNPs associated with *Ecklonia radiata* growth rate.** This table (included as an xlsx file) details all loci identified as significantly associated with growth rate using one or more complementary statistical methods. “ddRADtag” refers to the RAD-seq locus identifier and “SNP position” is the base-pair position within that tag. “Locus number” corresponds to the unique SNP index used in analyses and “R²_linear_model” gives the proportion of growth-rate variance explained by a single-locus linear model. “Method” indicates which approaches detected a significant association (GWAS, LFMM, and/or RDA).

Columns “er_genome_hit” and “linked_gene_model” indicate whether the ddRADtag aligns to the *E. radiata* draft genome and, if so, the nearest predicted gene model. “model_annot_ecarp_blastx_top5hits” lists the top *BLASTX* matches (when present) against the *Ectocarpus* protein database. “stack_in_transcriptome” indicates whether the ddRADtag aligns to the *E. radiata* transcriptome (Sequence Read Archive: SRR3709347) and “stack_transcript_match” gives percent identity of that match. “model_in_transcript” and “model_transcript_match” provide analogous transcriptome-alignment information for linked genome models (if present). “model_sequence” and “stack_sequence” contain nucleotide sequences of the annotated gene model and ddRADtag, respectively. Together, these metadata provide genomic context (coding vs. non-coding), transcriptomic evidence (gene expression support), and putative functional annotation for all loci associated with growth-rate variation in *E. radiata*.

**Supplementary Figures**


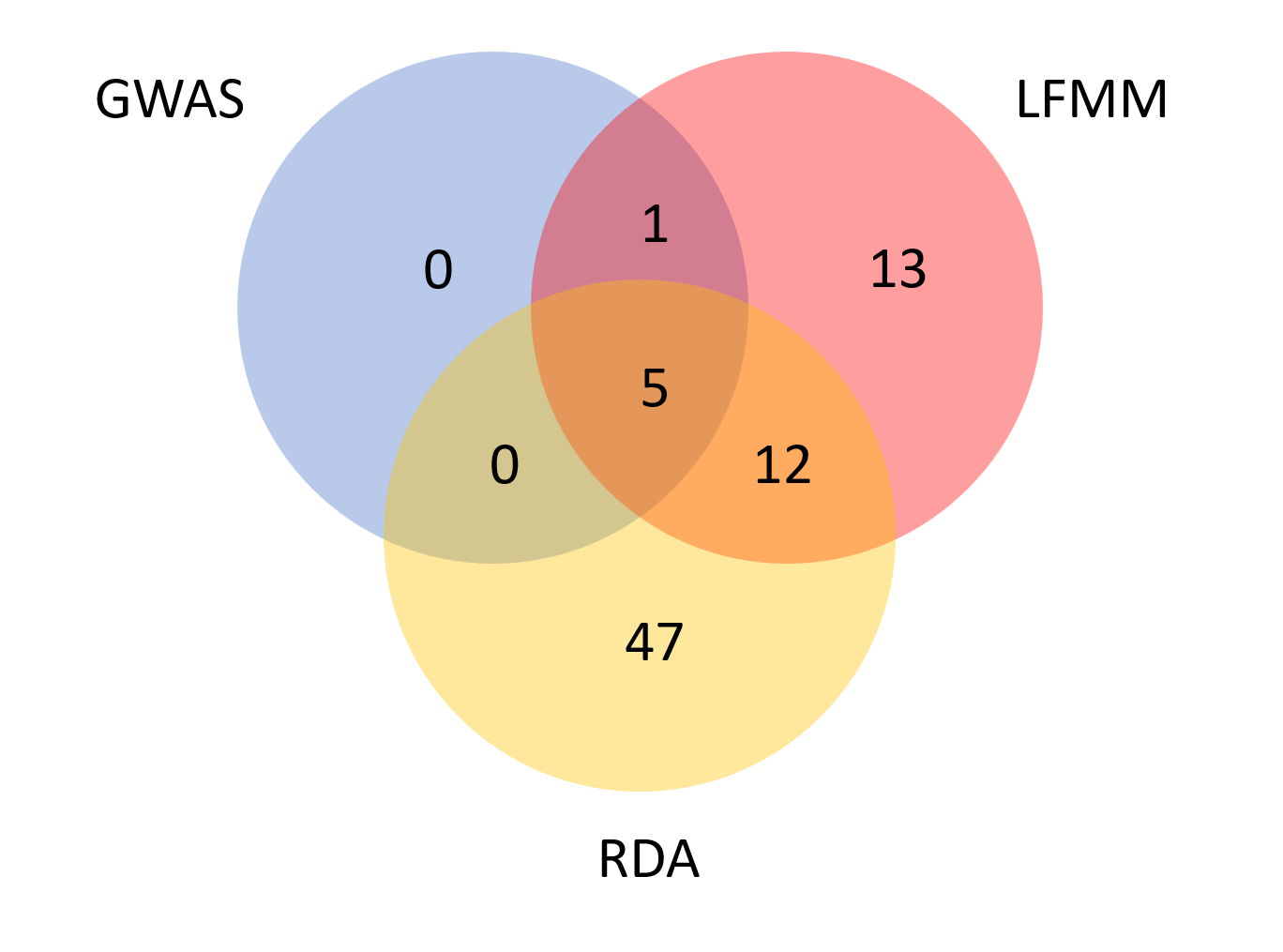


**Fig S1. Venn diagram showing number of significant loci detected with each method**. Note that 5 loci were significant across all three methods.


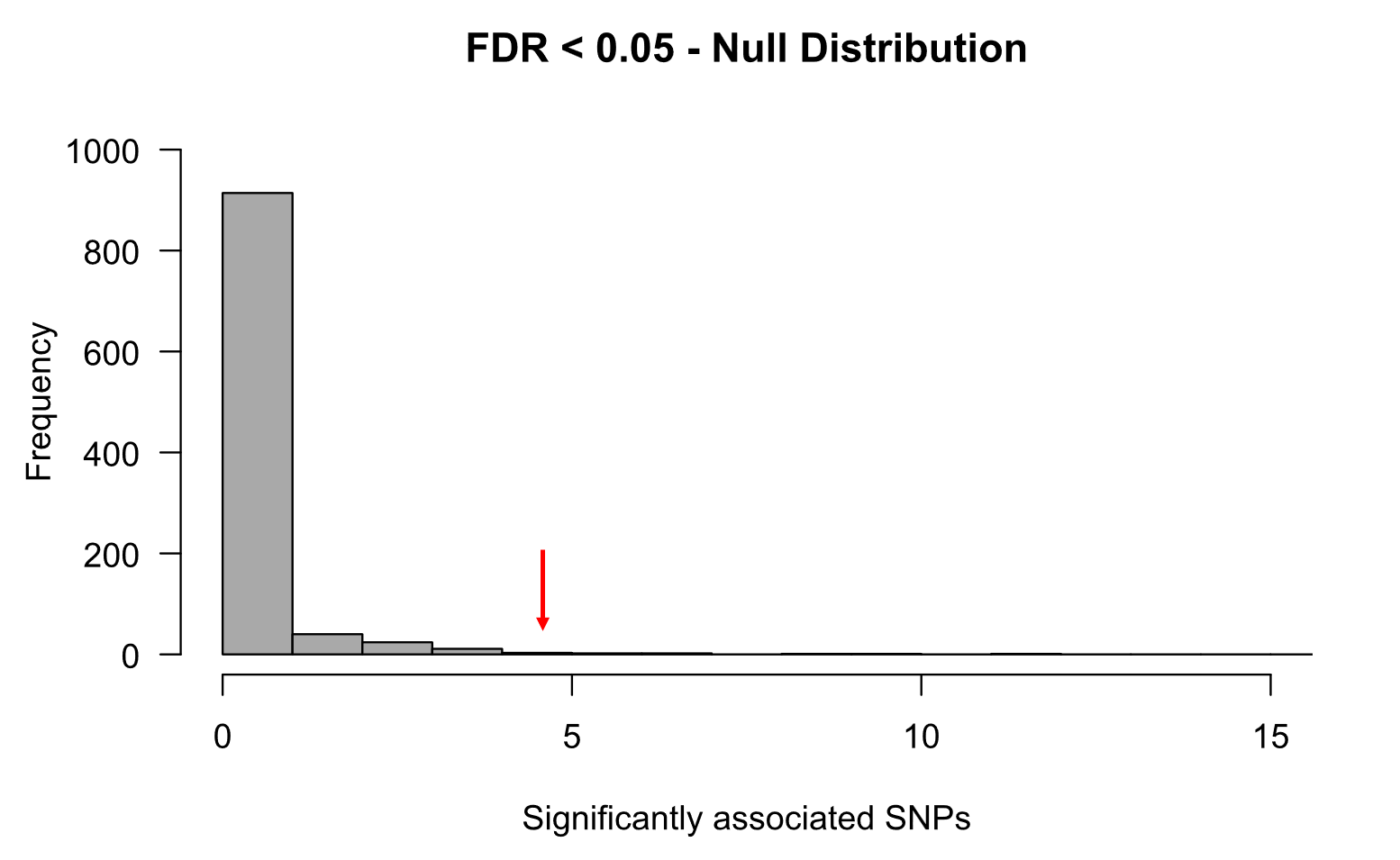


**Fig S2. Results of randomization test to determine probability of detecting outliers under a null hypothesis.** The histogram shows the frequency of identifying a certain number of significant associations by random chance. The number of significant GWAS associations (FDR < 0.05) detected in our observational data are shown with the red arrow.

**
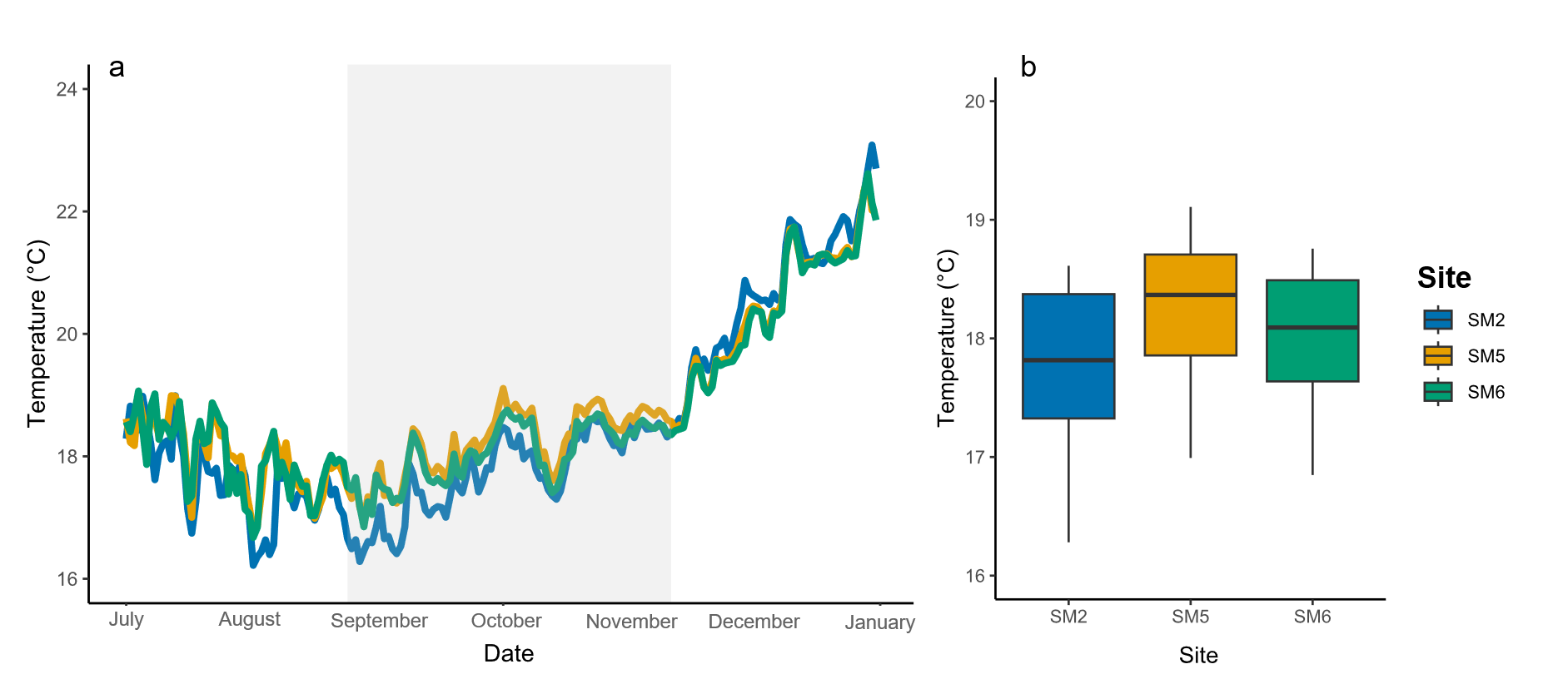
**

**Fig S3. In situ temperature measurements taken at the 3 sites included in this study.** (a) Temperature measurements at canopy height across all three sites. The shaded area indicates the window across which growth was measured (August to November). (b) Boxplot showing distributions of temperature measurements taken from each site across the measured growth window (shaded area in panel a).


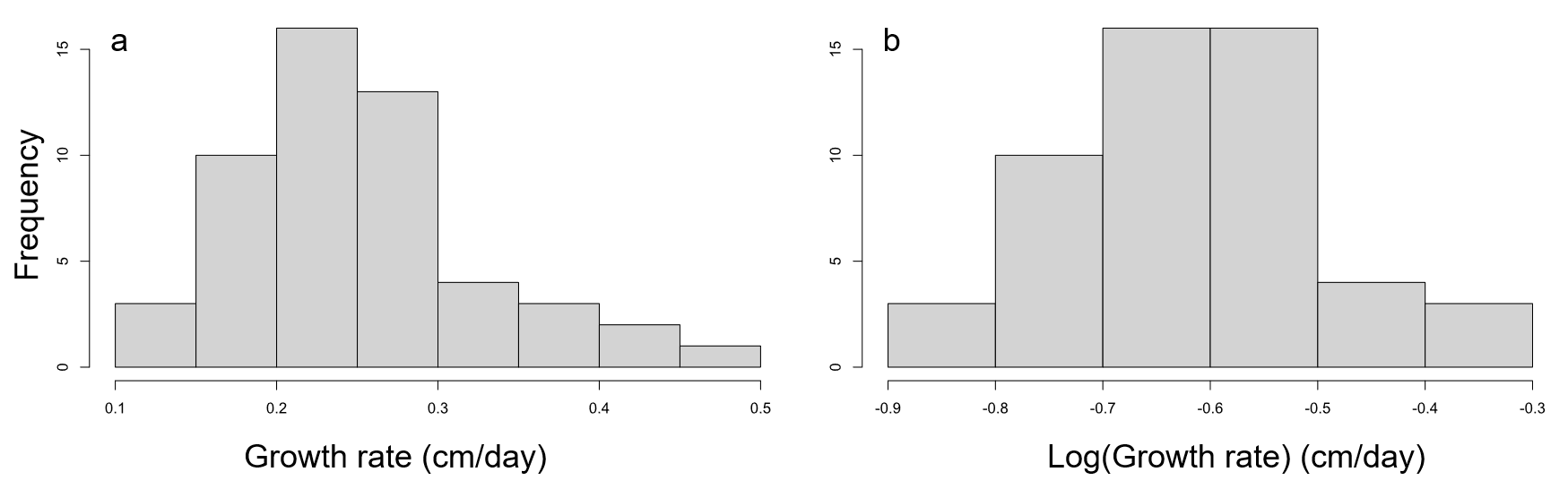


**Fig S4. Distribution of growth rate data before (a) and after (b) applying a log-transformation.** Note skew in non-transformed data.
